# Gene expression profiling in the developing secondary palate in the absence of *Tbx1* function

**DOI:** 10.1186/s12864-018-4782-y

**Published:** 2018-06-04

**Authors:** Maria Zoupa, Guilherme Machado Xavier, Stephanie Bryan, Ioannis Theologidis, Matthew Arno, Martyn T. Cobourne

**Affiliations:** 1 Centre for Craniofacial Development and Regeneration, King’s College London Dental Institute, Floor 27, Guy’s Tower, London, SE1 9RT UK; 2 Department of Orthodontics, King’s College London Dental Institute, London, UK; 3 Division of Development and Gene Expression, Institute of Molecular Biology and BiotechnologyFoundation for Research & Technology, Crete, Greece; 4 Genomics Centre, King’s College London, London, UK

**Keywords:** Palatogenesis, Cleft palate, Microarray, 22q11.2DS, DiGeorge syndrome

## Abstract

**Background:**

Microdeletion of chromosome 22q11 is associated with significant developmental anomalies, including disruption of the cardiac outflow tract, thymic/parathyroid aplasia and cleft palate. Amongst the genes within this region, *TBX1* is a major candidate for many of these developmental defects. Targeted deletion of *Tbx1* in the mouse has provided significant insight into the function of this transcription factor during early development of the cardiac and pharyngeal systems. However, less is known about its role during palatogenesis. To assess the influence of *Tbx1* function on gene expression profile within the developing palate we performed a microarray screen using total RNA isolated from the secondary palate of E13.5 mouse embryos wild type, heterozygous and mutant for *Tbx1*.

**Results:**

Expression-level filtering and statistical analysis revealed a total of 577 genes differentially expressed across genotypes. Data were clustered into 3 groups based on comparison between genotypes. Group A was composed of differentially expressed genes in mutant compared to wild type (*n* = 89); Group B included differentially expressed genes in heterozygous compared to wild type (*n* = 400) and Group C included differentially expressed genes in mutant compared to heterozygous (*n* = 88). High-throughput quantitative real-time PCR (RT-PCR) confirmed a total of 27 genes significantly changed between wild type and mutant; and 27 genes between heterozygote and mutant. Amongst these, the majority were present in both groups A and C (26 genes). Associations existed with hypertrophic cardiomyopathy, cardiac muscle contraction, dilated cardiomyopathy, focal adhesion, tight junction and calcium signalling pathways. No significant differences in gene expression were found between wild type and heterozygous palatal shelves.

**Conclusions:**

Significant differences in gene expression profile within the secondary palate of wild type and mutant embryos is consistent with a primary role for *Tbx1* during palatogenesis.

**Electronic supplementary material:**

The online version of this article (10.1186/s12864-018-4782-y) contains supplementary material, which is available to authorized users.

## Background

22q11.2 deletion syndrome (22q11.2DS) is the most common human microdeletion [1] occurring with a prevalence of 1:4000 and incidence ranging from 1:2000–6395 [2–4]. This microdeletion is associated with several syndromic conditions including DiGeorge (DGS; MIM 188400), velocardiofacial (VCFS; MIM 192430), conotruncal anomaly face (CAFS or Takao syndrome; MIM 217095) and isolated outflow tract (OFT) defects of the heart [5–9]. These conditions are characterized predominantly by the presence of congenital heart defects, thymic and parathyroid hypoplasia, and craniofacial dysmorphism, including oro-facial clefting that predominates as isolated cleft palate, micrognathia and (less commonly) dental defects [10–13]. The most common deletions are phenotypically indistinguishable from each other and consist of either a 3 Mb segment spanning the low copy repeats (LCR) A-D (around 85% of cases); or a smaller 1.5 Mb deletion that spans LCR A-B seen in around 15% of cases [14–16]. A less common LCR C-D deletion of the typical 22q11.2DS region has also been identified, which is associated with a much-reduced prevalence of cardiac malformations and oro-facial clefting [17–19]. 22q11.2DS is a contiguous gene and haploinsufficient syndrome with at least 30 different genes potentially contributing to the characteristic clinical features [20, 21]. Amongst the genes identified as candidates for the development of 22q11.2DS, T-Box 1 (*TBX1*), which encodes a T-Box-containing transcription factor is recognised as a major determinant through its location within the 22q11 critical region [21–23], expression in organs affected within the clinical spectrum [24–27] and observations that loss of *Tbx1* function in mouse recapitulates the clinical findings seen in many DGS subjects [23, 28–31]. Supporting this, *TBX1* mutation has been identified in a sporadic case of DGS [32] and *Tbx1* haploinsufficiency results in the most characteristic phenotypes related to developmental defects in the embryonic pharyngeal apparatus [32, 33]. DGS is also referred to as the III-IV pharyngeal pouch syndrome, as the pharyngeal pouches and their associated blood vessels are the structures most commonly affected [23, 30]. Apart from the aortic arch, thymus and parathyroid gland defects, *Tbx1* murine models also manifest craniofacial anomalies that arise from developmental defects associated with pharyngeal arches I and II [23, 34, 35]. Indeed, conditional mutant models have revealed a tissue-specific requirement and a dose sensitivity for *Tbx1* during murine pharyngeal development [20, 36–38].

The majority of 22q11.2DS individuals have a characteristic craniofacial morphology including lateral displacement of the inner canthi, swollen eyelids, small mouth, hypoplastic mandible, flat nasal bridge and square nose [39–41]. Cleft palate (including submucous cleft) is also present in approximately 10% of subjects [40]. Morphological studies to assess embryonic malformations in various *Tbx1* genotypes also reveal the presence of cleft palate in *Tbx1*-overexpressing mice [42, 43]. Therefore, both loss and gain of *Tbx1* function can lead to the development of a cleft phenotype.

The palate is divided anatomically into primary and secondary regions with the secondary palate composed of both hard and soft tissues. Embryologically, the secondary palate is derived from the paired maxillary processes of pharyngeal arch I, which gives rise to the palatal shelves. During palatogenesis, these shelves are initially situated bilaterally adjacent to the developing tongue; however, progressive growth and elevation results in them positioning themselves above the tongue, with further medial growth leading to fusion with their counterpart along the midline to create a single continuous palate. The palatal shelves also fuse with the nasal septum superiorly and primary palate anteriorly, completing separation of the nasal and oral cavities [44–46]. In the developing mouse embryo, *Tbx1* is expressed in epithelium of the palatal shelves throughout palatogenesis from embryonic day (E)12.5–15.5 [24]. The etiological basis of the cleft palate phenotype in *Tbx1* mutants is not fully understood but has been associated with abnormal palatal shelf elevation, possibly due to a combination of increased tongue height, decreased palatal shelf width, perturbed cell proliferation and apoptosis [47]. In addition, inappropriate fusion between the palatal shelf epithelium and tongue has also been described in this mutant, associated with hyper-proliferation and disrupted differentiation [48]. More recently, confocal image analysis has found only subtle differences in levels of proliferation within mesenchyme of the palatal shelves between wild-type and mutant until the later stages of palatogenesis; although significant differences in mesenchymal cell orientation were found in mutant shelves, which might contribute to the cleft phenotype [49].

We are interested in further defining the role of *Tbx1* during the process of murine palatogenesis. Specifically, we have investigated regulation of this transcription factor in the secondary palate and carried out a functionally-based microarray using the *Tbx1* mouse model. We compared total RNA isolated from dissected secondary palatal shelves derived from E13.5 wild type (WT), Tbx1^+/−^ (heterozygous) and *Tbx1*^*−/−*^ (mutant) embryos and clustered the data into three groups based on comparison between the three genotypes. Microarray analysis demonstrated that in the absence of functional *Tbx1*, significant changes occur in the expression profile of numerous genes in mutant versus WT and mutant versus heterozygous groups. The most significant pathways affected in both groups were the hypertrophic cardiomyopathy, cardiac muscle contraction, dilated cardiomyopathy, focal adhesion, calcium signalling and tight junction pathways. High-throughput quantitative RT-PCR validation confirmed significant variation between WT and mutant in the expression of 26 individual genes. We discuss these findings within the context of murine secondary palatogenesis.

## Results

### Regulation of Tbx1 in the developing secondary palate

**Fig. 1:**
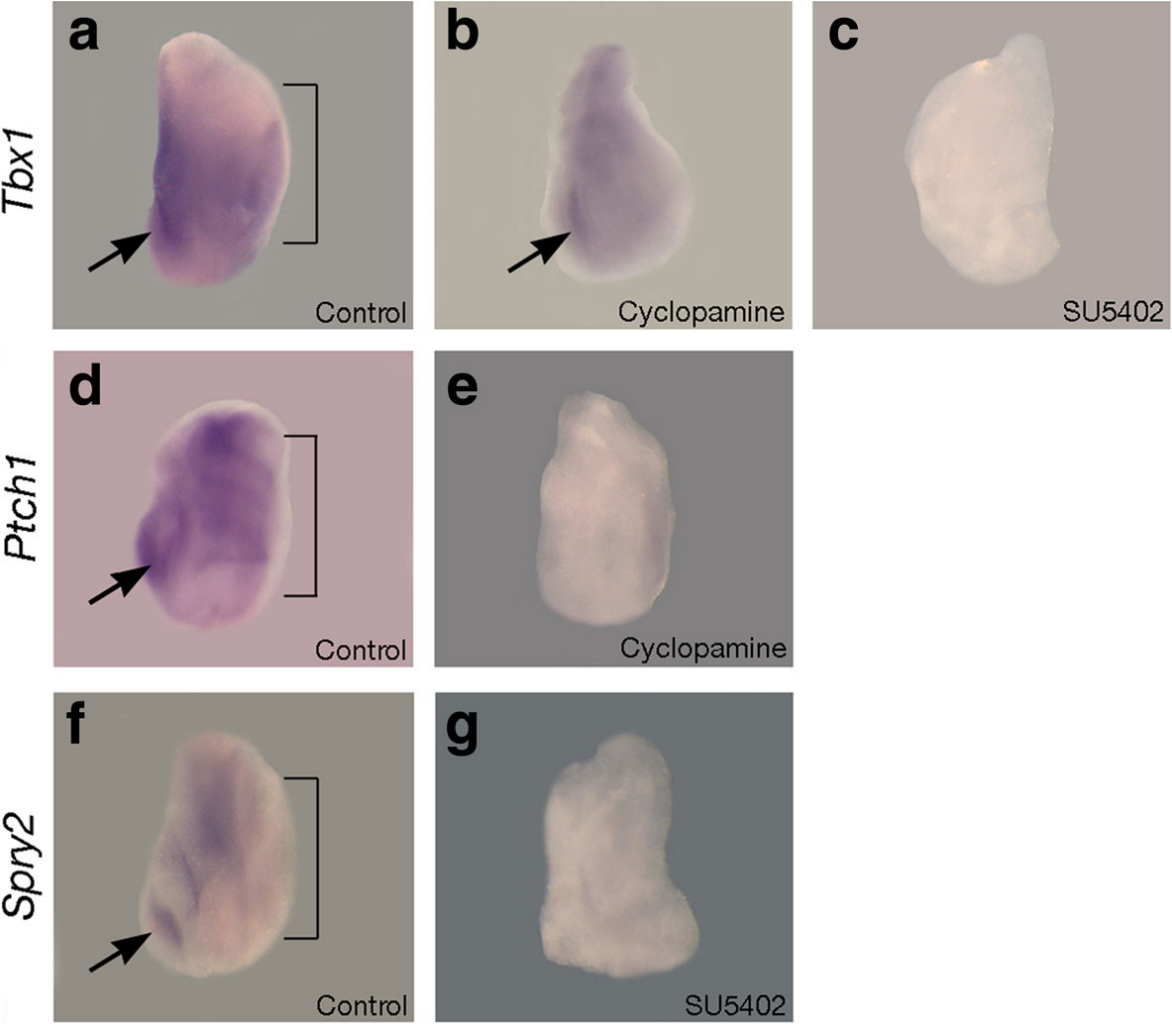
Regulation of *Tbx1* expression in the early secondary palate. Wholemount in situ hybridization on palatal shelf explants cultured for 24 h in the presence or absence of the Shh inhibitor cyclopamine and the Fgf receptor inhibitor SU5402. **a** *Tbx1* is expressed in the palatal shelf epithelium and first molar tooth germ (arrowed); (**b**) in the absence of Shh signaling, *Tbx1* is maintained; (**c**) in the absence of Fgf signaling, *Tbx1* is lost; (**d**) Shh signaling is active in the developing palate and first molar (arrowed) as shown by expression of the Shh transcriptional target patched1 (*Ptch1*); (**e**) in the presence of cyclopamine *Ptch1* transcription is lost; (**f**) Fgf signaling is active in the developing palate and first molar (arrowed), as shown by expression of the Fgf transcriptional target sprouty2 (*Spry2*); (**g**) in the presence of SU4502 *Spry2* is lost. Lines mark the medial edge of the palatal shelf

*Tbx1* transcriptional activity is present in epithelium of the secondary palate shelves throughout the processes of growth, elevation and fusion (Additional file 1) and *Tbx1* mutant mice have a fully penetrant cleft palate [23, 30, 31]. We are interested in further defining the function of this transcription factor during palatogenesis at the molecular level and first sought to understand how *Tbx1* transcription might be regulated in the palatal shelf epithelium. We began by investigating the effect of abrogating either Sonic hedgehog (Shh) or Fibroblast growth factor (Fgf) signaling in palatal shelf explants as there are potential associations between these signaling networks and Tbx1 function in the developing palate. *Shh* is also expressed in the palatal epithelium and lies upstream of *Tbx1* in the pharyngeal endoderm [50]; whilst Fgf signaling can maintain *Tbx1* expression in early odontogenic epithelium [27]. Specifically, E13.5 secondary palatal shelves were isolated and cultured for 24 h in the presence of either the Shh antagonist cyclopamine or the Fgf receptor inhibitor SU4502. Interestingly, whilst an absence of Shh signaling did not affect *Tbx1* transcription, loss of Fgf signaling resulted in a loss of *Tbx1* activity in the palatal epithelium after 24 h of culture (Fig. 1a-g). These results place *Tbx1* downstream of Fgf signaling during early palatogenesis and in contrast to the pharyngeal region, loss of Shh does not affect *Tbx1*.

### Altered gene expression in the secondary palate of Tbx1 mutant mice

It is known that Shh, Fgf and Bone morphogenetic protein (Bmp) signaling pathways are important during normal development of the palate [51–53]; in particular, reciprocal signaling between epithelial Shh and mesenchymal *Fgf10*, mediated through fibroblast growth factor receptor 2b (Fgfr2b), regulates cell proliferation in the mesenchyme [54]. Whilst Shh also negatively regulates *Bmp4* in the mesenchyme, which is itself upstream of Fgf10 [55]. Tbx1 interacts with a number of these molecules during embryogenesis, being directly upstream of *Fgf10* in the early heart field [28, 56]; negatively modulating Bmp4 through the binding of Smad1 in cardiomyocytes [36] and being downstream of Shh in endoderm of the early pharynx [50]. Within the palate itself, it has been variously suggested that Tbx1 negatively regulates *Fgf10* and *Bmp4*, whilst positively regulating *Fgf8* and *Pax9*, although there is currently not a consensus on these findings [47, 48].

**Fig. 2:**
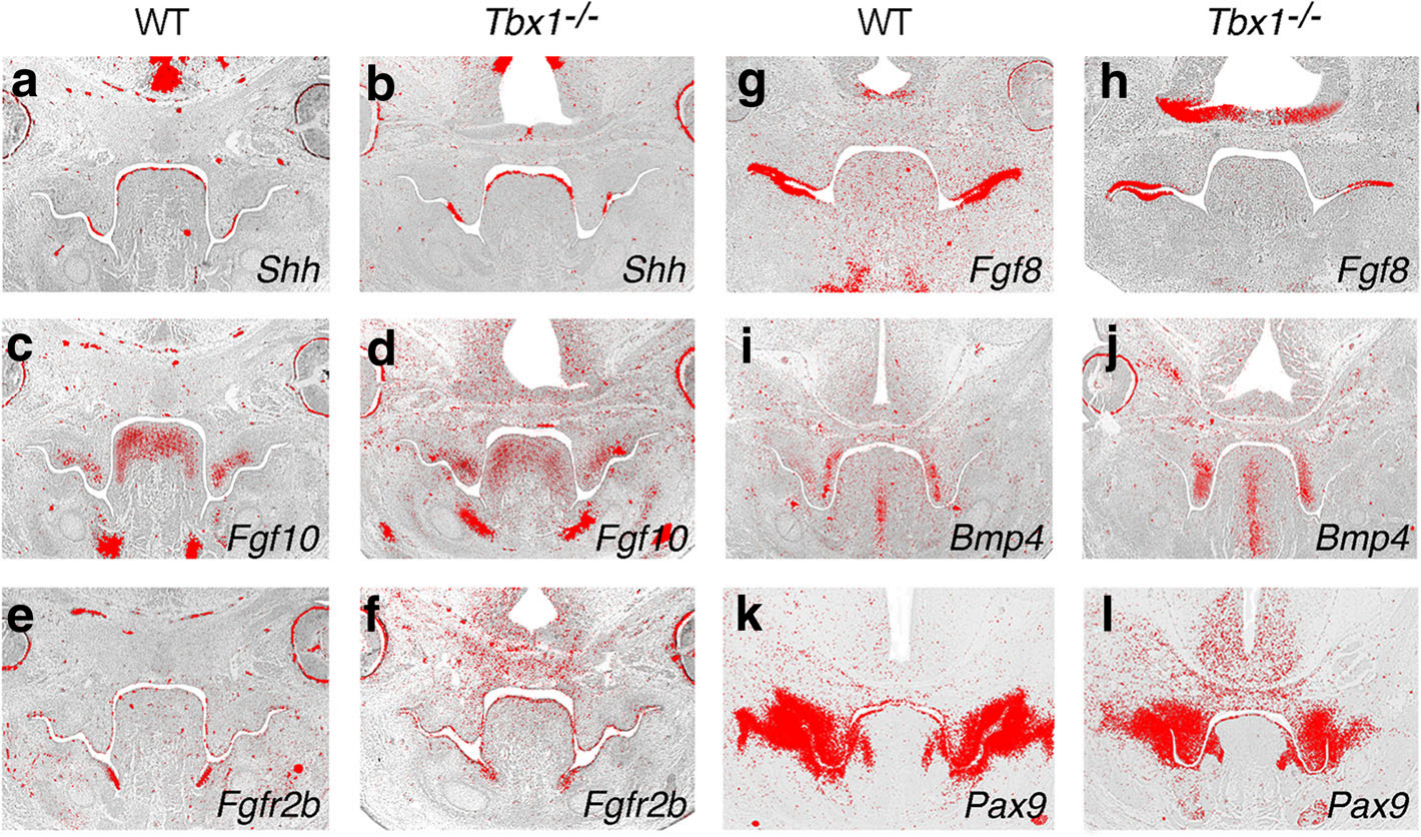
Signaling interactions during development of the secondary palate in WT and *Tbx1* mutant embryos. Section in situ hybridization demonstrating the expression of key signaling molecules. **a**, **b** *Shh*; (**c**, **d**) *Fgf10*; (**e**, **f**) *Fgfr2b*; (**g**, **h**) *Fgf8*; (**i**, **j**) *Bmp4*; (**k**, **l**) *Pax9*

Although we could find no evidence that *Tbx1* is downstream of Shh signaling in the palatal epithelium, there is considerable overlap of expression. We therefore investigated known targets of Shh within palatal shelves WT and mutant for *Tbx1* using in situ hybridization. Interestingly, we found no significant differences in expression of *Shh*, *Fgf10* and *Fgfr2b* between WT and mutant (Fig. 2a-f). However, whilst *Fgf8* expression was also normal in the mutant shelves (Fig. 2g-h), *Bmp4* and paired-box 9 (*Pax9)* were slightly up and downregulated, respectively in the posterior region of the secondary palate (Fig. 2i-l). These apparent changes in *Bmp4* and *Pax9* expression in the mutant might simply be a function of altered numbers of cells expressing these genes in the palate mesenchyme, particularly as the *Tbx1* domain within the palatal epithelium does not completely overlie those of *Bmp4* or *Pax9* in the mesenchyme [48]. However, given the evidence of retarded growth in *Tbx1* mutant palatal shelves [47, 48] if an alteration in cell number is responsible for any of these changes, it would seem to be more likely for *Pax9*.

### Microarray analysis

To further identify potential transcriptional target genes of Tbx1 implicated in palatogenesis, microarray analysis was carried out using cDNA transcribed from total RNA derived from the dissected secondary palatal shelves of E13.5 *Tbx1*^*+/+*^; *Tbx1*^*+/−*^ and *Tbx1*^*−/−*^ embryos (*n* = 3 for each genotype).

**Fig. 3:**
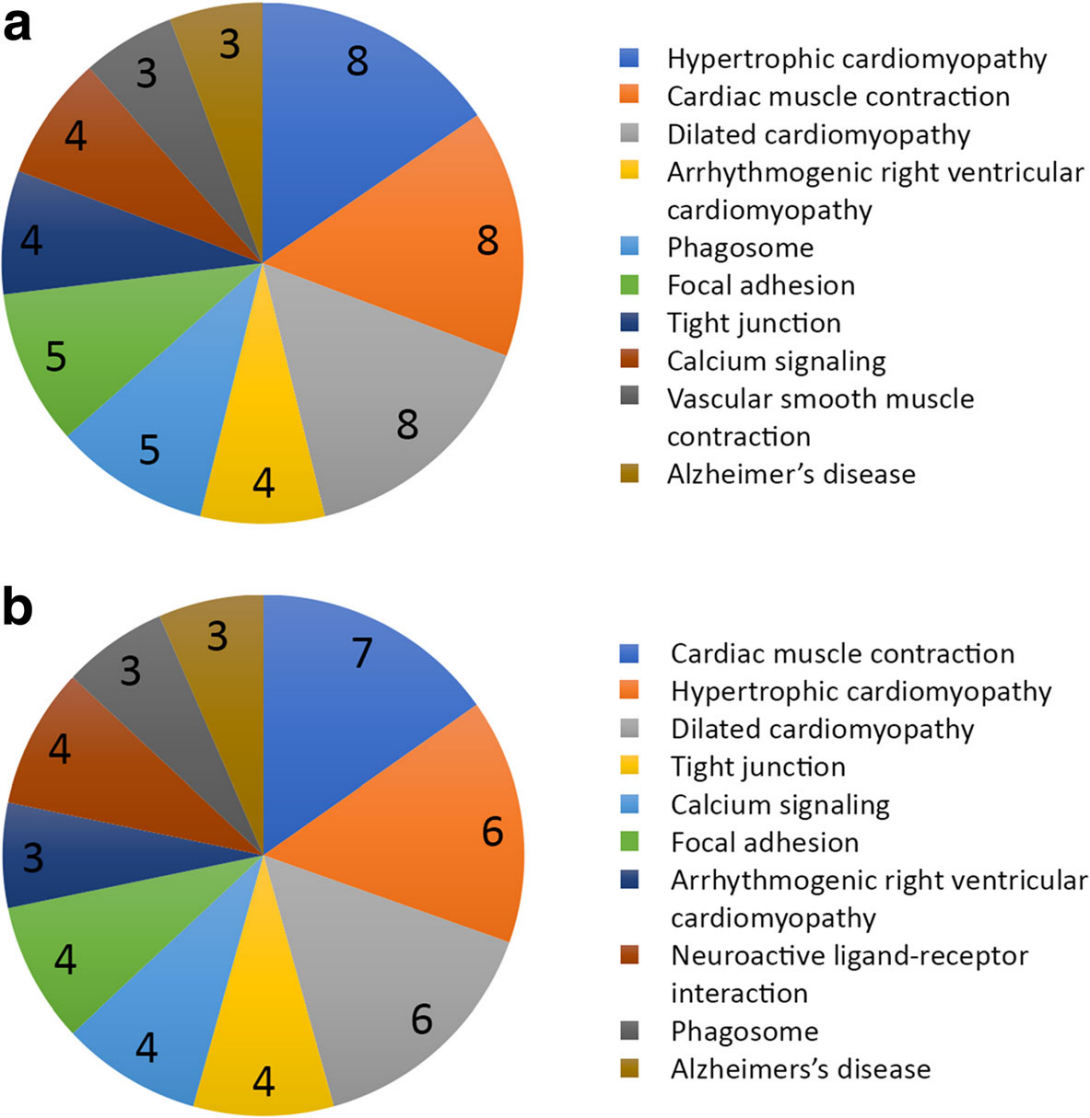
**a** Pathway analysis of genes differentially expressed in the *Tbx1* mutant secondary palate compared to WT (Group A); (**b**) pathway analysis of genes differentially expressed in the mutant secondary palate when compared to heterozygous (Group C): The pie chart depicts the number of assigned genes for each significantly enriched pathway. Data sets are illustrated as slices, the sizes of which are proportional to the number of genes implicated in each pathway. The ten pathways are listed and colour-coded on the right

**Fig. 4:**
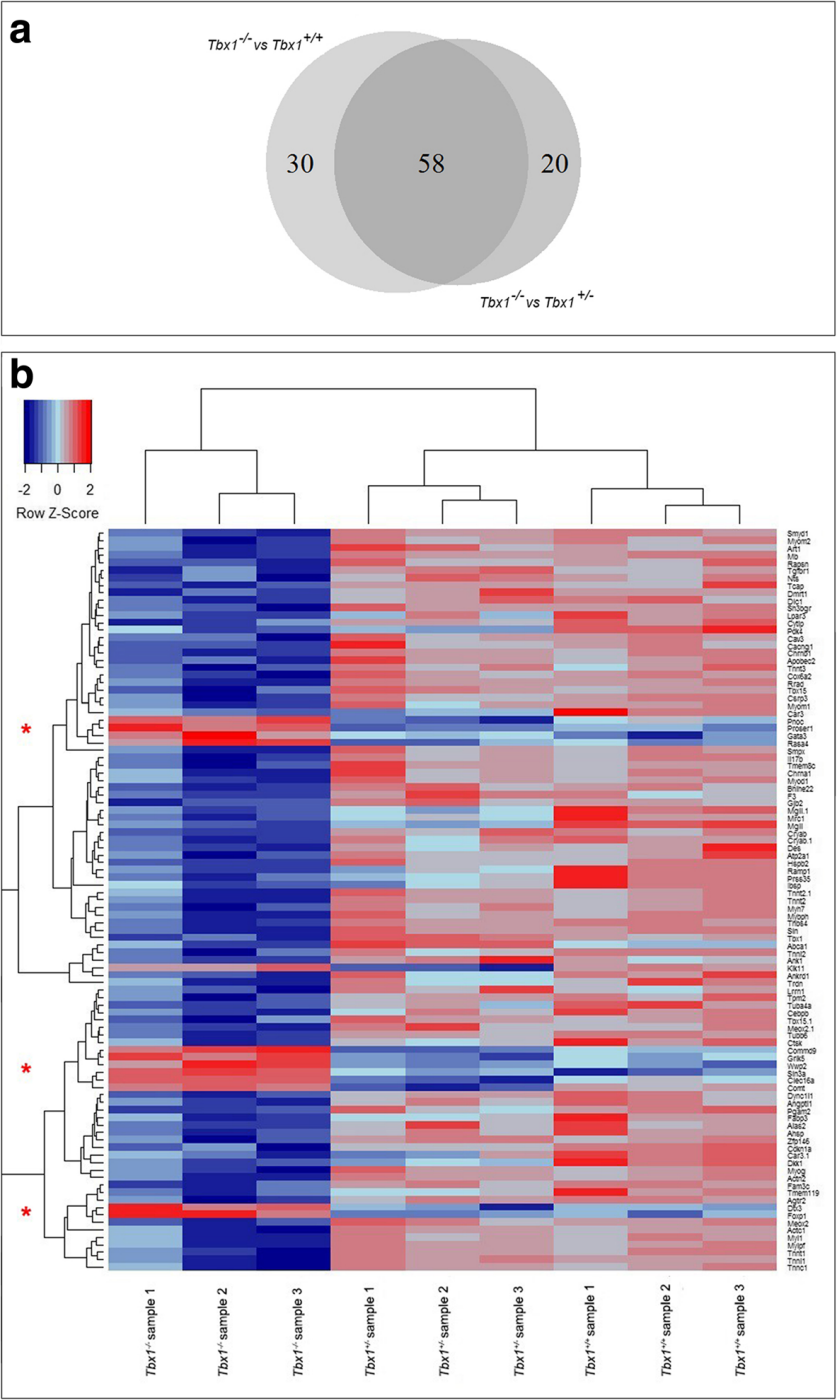
**a** Pairwise Venn diagram illustrating the comparison between gene sets from *Tbx1* mutant secondary palate compared to WT (Group A) and *Tbx1* mutant compared to heterozygous (Group C). The Venn diagram identified 58 common elements between Group A and Group C. Numbers in each section represent the number of genes. Transcripts utilized for the construction of the Venn diagram were statistically significant with adj. *p* values < 0.1; (**b**) heat map (hierarchical clustering) of commonly expressed genes in Groups A and C, as well as uniquely expressed genes in Group A and C. Hierarchical cluster of 99 genes found to be differentially expressed in the 3 mutant, 3 heterozygous and 3 WT palatal samples. Transcripts utilized for the construction of clustering were statistically significant with adj. *p* values < 0.1 except for heterozygous where adj. *p* values were > 0.1. Visual inspection of heat map and dendogram clustering of the 9 samples revealed that all triplicates of the same genotype clustered together. Upregulated genes in mutants clustered together (red asterisks on left) and their pattern of expression could be visibly compared top heterozygous and WT samples. Each row represents a specific gene, and each column represents each genotype of the samples analysed. The colour represents the expression level of the gene. Red represents high expression, while blue represents low expression. The expression levels are continuously mapped on the colour scale provided at the top left of the figure. The dendrogram at the top of the matrix provides the degree of similarity between examined groups assessing the similarity between expressed genes and samples used for comparison. Note the similarity in gene expression between WT and *Tbx1* heterozygous transcripts

After normalization and filtering of microarray data, comparison between mutant embryos and WT (Group A), heterozygous and WT (Group B) and mutant versus heterozygous (Group C) were performed (adj. *p* < 0.1). The WebGestalt database was used to identify biological pathways associated with these differentially expressed transcripts [57]. In Group A, 89 genes were identified to be differentially expressed in mutant compared to WT (adj. *p* < 0.1, fold change 1.4). From these, 3 genes were upregulated, whereas the majority (*n* = 86) were downregulated (Table 1). Group B includes differentially expressed genes arising from the comparison of heterozygous and WT palates (*n* = 400, adj. *p* > 0.23). This group list was not considered statistically significant (adj. *p* > 0.1) and therefore was not analysed further (Additional file 2). In Group C, 88 genes were identified to be differentially expressed in the mutant compared to heterozygote palate (adj. *p* < 0.1, fold change 1.3). Amongst these, 11 genes were upregulated, whereas 77 were downregulated (Table 1). In Group A, from the 89 genes that were searched, 9 Kyoto Encyclopedia of Genes and Genomes (KEGG) pathways were identified (Fig. 3a). The most statistically enriched pathways (adj. *p* < 0.1) were all associated with cardiac muscle physiology and included hypertrophic cardiomyopathy, cardiac muscle contraction, dilated cardiomyopathy, arrhythmogenic right ventricular cardiomyopathy and vascular smooth muscle contraction. Other pathways included phagosome and focal adhesion, tight junction and calcium signaling pathways and Alzheimer's disease (Additional file 3). In Group C, from the 88 genes that were searched, 10 KEGG pathways were identified (Fig. 3b). The most statistically enriched pathways (adj. *p* < 0.1) were all also associated with cardiac muscle physiology, including hypertrophic and dilated cardiomyopathy and arrhythmogenic right ventricular cardiomyopathy. Other pathways included tight junction, calcium signalling, focal adhesion, neuroactive ligand-receptor interaction, phagosome and Alzheimer’s disease pathways (Additional file 3). We were then interested to further identify the proportion of overlap amongst significantly differentially expressed genes between Groups A and C. (Fig. 4a [58]). The two groups share 58 commonly expressed genes (Table 2) when compared to WT and heterozygous; whereas 30 genes (Table 2) were uniquely observed in Group A and 20 in Group C (Table 2; adj. *P* < 0.1). The WebGestalt database was used to provide insights into the mechanism of regulation associated with these 58 common gene transcripts. Adding to the above approach, heat map and dendogram clustering of the commonly expressed genes, as well as uniquely expressed genes in Group A and Group C (*n* = 99 genes) revealed transcriptional homogenicity between genotypes (Fig. 4b). Genes upregulated in mutants clearly clustered together and were shown to be downregulated in heterozygote and WT samples (red asterisks in Fig. 4b). In contrast, the downregulated transcriptome of mutant samples was shown to increase its expression in heterozygous and WT palates. Although statistical analysis revealed a non-significant expression pattern of Tbx1 heterozygous samples (adj. *p* values > 0.1), heat map revealed a similarity in gene expression pattern between heterozygous and WT samples.

**Table 1:** Group comparison of *Tbx1*^*+/+*^, *Tbx1*^*+/−*^ and *Tbx1*^*−/−*^ palatal shelves

Gene ID	Gene symbol	Description	logFC	Fold Change
Group A: Genes differentially expressed in mutant compared to WT palates				
14,462	*Gata3*	GATA binding protein 3	1,10	2,15
66894	*Wwp2*	WW domain containing E3 ubiquitin protein ligase 2	0,64	1,55
20466	*Sin3a*	transcriptional regulator, SIN3A (yeast)	0,45	1,37
27999	*Fam3c*	family with sequence similarity 3, member C	−0,43	−1,35
23,945	*Mgll*	monoglyceride lipase	−0,44	−1,36
22145	*Tuba4a*	tubulin, alpha 4A	−0,46	−1,38
23,945	*Mgll*	monoglyceride lipase	−0,46	−1,38
17286	*Meox2*	mesenchyme homeobox 2	− 0,48	− 1,39
227929	*Cytip*	cytohesin 1 interacting protein	−0,50	−1,41
21393	*Tcap*	titin-cap	−0,50	−1,42
13426	*Dync1i1*	dynein cytoplasmic 1 intermediate chain 1	−0,51	−1,42
231,633	*Tmem119*	transmembrane protein 119	−0,52	−1,43
21953	*Tnni2*	troponin I, skeletal, fast 2	−0,54	−1,46
27,273	*Pdk4*	pyruvate dehydrogenase kinase, isoenzyme 4	−0,54	−1,46
13,038	*Ctsk*	cathepsin K	−0,57	− 1,48
107765	*Ankrd1*	ankyrin repeat domain 1 (cardiac muscle)	−0,57	−1,49
17533	*Mrc1*	mannose receptor, C type 1	−0,59	−1,50
50796	*Dmrt1*	doublesex and mab-3 related transcription factor 1	−0,59	−1,51
72713	*Angptl1*	angiopoietin-like 1	−0,61	−1,53
13346	*Des*	desmin	−0,67	−1,59
12862	*Cox6a2*	cytochrome c oxidase subunit VIa polypeptide 2	−0,69	−1,61
56437	*Rrad*	Ras-related associated with diabetes	−0,71	−1,64
12608	*Cebpb*	CCAAT/enhancer binding protein (C/EBP), beta	−0,71	−1,64
14066	*F3*	coagulation factor III	−0,74	−1,67
50768	*Dlc1*	deleted in liver cancer 1	−0,74	− 1,67
12299	*Cacng1*	calcium channel, voltage-dependent, gamma subunit 1	−0,74	−1,67
76,757	*Trdn*	triadin	−0,76	−1,69
11475	*Acta2*	actin, alpha 2, smooth muscle, aorta	−0,76	− 1,69
12292	*Cacna1s*	calcium channel, voltage-dependent, L type, alpha 1S subunit	−0,76	−1,70
56012	*Pgam2*	phosphoglycerate mutase 2	−0,79	−1,73
67951	*Tubb6*	tubulin, beta 6 class V	−0,83	−1,78
11656	*Alas2*	aminolevulinic acid synthase 2, erythroid	−0,84	− 1,80
19400	*Rapsn*	receptor-associated protein of the synapse	−0,85	−1,80
22004	*Tpm2*	tropomyosin 2, beta	−0,86	−1,82
12575	*Cdkn1a*	cyclin-dependent kinase inhibitor 1A (P21)	−0,87	−1,83
17189	*Mb*	myoglobin	−0,88	−1,85
11609	*Agtr2*	angiotensin II receptor, type 2	−0,90	−1,86
21384	*Tbx15*	T-box 15	− 0,91	− 1,87
12955	*Cryab*	crystallin, alpha B	−0,92	−1,89
12955	*Cryab*	crystallin, alpha B	−0,92	−1,89
50795	*Sh3bgr*	SH3-binding domain glutamic acid-rich protein	−0,92	−1,89
17930	*Myom2*	myomesin 2	−0,95	−1,93
12180	*Smyd1*	SET and MYND domain containing 1	−0,96	−1,94
59058	*Bhlhe22*	basic helix-loop-helix family, member e22	−0,96	−1,95
26465	*Zfp146*	zinc finger protein 146	−1,01	−2,01
12391	*Cav3*	caveolin 3	−1,02	−2,02
65086	*Lpar3*	lysophosphatidic acid receptor 3	−1,06	−2,09
170812	*Ahsp*	alpha hemoglobin stabilizing protein	−1,09	−2,13
14,077	*Fabp3*	fatty acid binding protein 3, muscle and heart	−1,10	−2,15
11443	*Chrnb1*	cholinergic receptor, nicotinic, beta polypeptide 1 (muscle)	−1,11	−2,16
17929	*Myom1*	myomesin 1	−1,14	−2,20
21953	*Tnni2*	troponin I, skeletal, fast 2	−1,16	−2,24
244954	*Prss35*	protease, serine 35	−1,19	−2,29
69253	*Hspb2*	heat shock protein 2	−1,20	−2,29
21957	*Tnnt3*	troponin T3, skeletal, fast	−1,23	−2,35
14619	*Gjb2*	gap junction protein, beta 2	−1,24	−2,36
13009	*Csrp3*	cysteine and glycine-rich protein 3	−1,30	−2,46
12,350	*Car3*	carbonic anhydrase 3	−1,37	−2,59
56069	*Il17b*	interleukin 17B	−1,37	−2,59
11811	*Apobec2*	apolipoprotein B mRNA editing enzyme, catalytic polypeptide 2	−1,43	−2,69
11937	*Atp2a1*	ATPase, Ca++ transporting, cardiac muscle, fast twitch 1	−1,46	−2,76
66139	*Tmem8c*	transmembrane protein 8C	−1,48	−2,78
51801	*Ramp1*	receptor (calcitonin) activity modifying protein 1	−1,56	−2,94
24131	*Ldb3*	LIM domain binding 3	−1,56	−2,94
16545	*Kera*	keratocan	−1,81	−3,51
140781	*Myh7*	myosin, heavy polypeptide 7, cardiac muscle, beta	−1,81	−3,51
21828	*Thbs4*	thrombospondin 4	−1,91	−3,75
13380	*Dkk1*	dickkopf homolog 1 (*Xenopus laevis*)	− 1,94	−3,83
21955	*Tnnt1*	troponin T1, skeletal, slow	−1,95	−3,87
58916	*Myot*	myotilin	−1,98	−3,95
17928	*Myog*	myogenin	−2,04	−4,12
21380	*Tbx1*	T-box 1	− 2,06	−4,16
53311	*Mybph*	myosin binding protein H	−2,06	−4,16
21952	*Tnni1*	troponin I, skeletal, slow 1	−2,26	−4,79
12,350	*Car3*	carbonic anhydrase 3	−2,31	−4,97
66402	*Sln*	sarcolipin	−2,40	−5,28
11472	*Actn2*	actinin alpha 2	−2,40	−5,29
17896	*Myl4*	myosin, light polypeptide 4	−2,44	−5,43
21956	*Tnnt2*	troponin T2, cardiac	−2,53	−5,77
11464	*Actc1*	actin, alpha, cardiac muscle 1	−2,56	−5,90
66106	*Smpx*	small muscle protein, X-linked	−2,61	−6,11
21924	*Tnnc1*	troponin C, cardiac/slow skeletal	−2,67	−6,35
17901	*Myl1*	myosin, light polypeptide 1	−2,76	−6,76
21925	*Tnnc2*	troponin C2, fast	−2,77	−6,83
17907	*Mylpf*	myosin light chain, phosphorylatable, fast skeletal muscle	−2,88	−7,35
21956	*Tnnt2*	troponin T2, cardiac	−2,92	−7,57
11459	*Acta1*	actin, alpha 1, skeletal muscle	−3,10	−8,60
17883	*Myh3*	myosin, heavy polypeptide 3, skeletal muscle, embryonic	−3,22	−9,29
15,891	*Ibsp*	integrin binding sialoprotein	−3,51	−11,36
Group C: Genes differentially expressed in mutant compared to heterozygous palates				
12,846	*Comt*	catechol-O-methyltransferase	1,0	2,1
74,374	*Clec16a*	C-type lectin domain family 16, member A	0,8	1,8
54153	*Rasa4*	RAS p21 protein activator 4	0,7	1,6
66894	*Wwp2*	WW domain containing E3 ubiquitin protein ligase 2	0,6	1,5
18,155	*Pnoc*	prepronociceptin	0,6	1,5
56,538	*Klk11*	kallikrein related-peptidase 11	0,5	1,4
80904	*Dtx3*	deltex 3 homolog (Drosophila)	0,5	1,4
212,127	*Proser1*	proline and serine rich 1	0,5	1,4
108655	*Foxp1*	forkhead box P1	0,4	1,4
76501	*Commd9*	COMM domain containing 9	0,4	1,4
14809	*Grik5*	glutamate receptor, ionotropic, kainate 5 (gamma 2)	0,4	1,3
19280	*Ptprs*	protein tyrosine phosphatase, receptor type, S	−0,3	−1,3
18,008	*Nes*	nestin	−0,4	−1,3
27999	*Fam3c*	family with sequence similarity 3, member C	−0,4	−1,3
13426	*Dync1i1*	dynein cytoplasmic 1 intermediate chain 1	−0,4	−1,3
65114	*Vps35*	vacuolar protein sorting 35	−0,5	−1,4
21393	*Tcap*	titin-cap	−0,5	−1,4
17286	*Meox2*	mesenchyme homeobox 2	− 0,5	− 1,4
17286	*Meox2*	mesenchyme homeobox 2	− 0,5	− 1,4
72713	*Angptl1*	angiopoietin-like 1	−0,5	−1,4
67405	*Nts*	neurotensin	−0,6	− 1,5
11,303	*Abca1*	ATP-binding cassette, sub-family A (ABC1), member 1	−0,6	−1,5
21812	*Tgfbr1*	transforming growth factor, beta receptor I	−0,6	−1,5
15,366	*Hmmr*	hyaluronan mediated motility receptor (RHAMM)	−0,6	−1,5
11,733	*Ank1*	ankyrin 1, erythroid	−0,6	−1,5
21412	*Tcf21*	transcription factor 21	−0,6	−1,5
50796	*Dmrt1*	doublesex and mab-3 related transcription factor 1	−0,7	−1,6
12862	*Cox6a2*	cytochrome c oxidase subunit VIa polypeptide 2	−0,7	−1,6
50768	*Dlc1*	deleted in liver cancer 1	−0,7	−1,6
56437	*Rrad*	Ras-related associated with diabetes	−0,7	−1,6
56012	*Pgam2*	phosphoglycerate mutase 2	−0,7	−1,6
67951	*Tubb6*	tubulin, beta 6 class V	−0,7	−1,6
11,870	*Art1*	ADP-ribosyltransferase 1	−0,7	−1,7
15375	*Foxa1*	forkhead box A1	−0,7	−1,7
11,475	*Acta2*	actin, alpha 2, smooth muscle, aorta	−0,8	−1,7
12292	*Cacna1s*	calcium channel, voltage-dependent, L type, alpha 1S subunit	−0,8	−1,7
19400	*Rapsn*	receptor-associated protein of the synapse	−0,8	−1,7
80,882,479	*Lrrn1*	leucine rich repeat protein 1, neuronal	−0,8	−1,7
17189	*Mb*	myoglobin	−0,8	−1,7
12299	*Cacng1*	calcium channel, voltage-dependent, gamma subunit 1	−0,8	−1,8
12955	*Cryab*	crystallin, alpha B	−0,8	−1,8
11609	*Agtr2*	angiotensin II receptor, type 2	−0,9	−1,8
111,886,114	*Cryab*	crystallin, alpha B	−0,9	−1,8
17930	*Myom2*	myomesin 2	−0,9	−1,8
12180	*Smyd1*	SET and MYND domain containing 1	−0,9	−1,8
170812	*Ahsp*	alpha hemoglobin stabilizing protein	−0,9	−1,9
50795	*Sh3bgr*	SH3-binding domain glutamic acid-rich protein	−0,9	−1,9
14066	*F3*	coagulation factor III	−0,9	−1,9
59058	*Bhlhe22*	basic helix-loop-helix family, member e22	−1,0	−2,0
12391	*Cav3*	caveolin 3	−1,0	−2,1
17929	*Myom1*	myomesin 1	−1,1	−2,1
26465	*Zfp146*	zinc finger protein 146	−1,1	−2,1
21384	*Tbx15*	T-box 15	− 1,1	−2,1
21384	*Tbx15*	T-box 15	− 1,1	−2,2
11443	*Chrnb1*	cholinergic receptor, nicotinic, beta polypeptide 1 (muscle)	−1,1	−2,2
21953	*Tnni2*	troponin I, skeletal, fast 2	−1,2	−2,2
69253	*Hspb2*	heat shock protein 2	−1,2	−2,2
13009	*Csrp3*	cysteine and glycine-rich protein 3	−1,2	−2,3
21957	*Tnnt3*	troponin T3, skeletal, fast	−1,3	−2,4
11937	*Atp2a1*	ATPase, Ca++ transporting, cardiac muscle, fast twitch 1	−1,3	−2,4
56069	*Il17b*	interleukin 17B	−1,3	−2,5
14619	*Gjb2*	gap junction protein, beta 2	−1,5	−2,8
11435	*Chrna1*	cholinergic receptor, nicotinic, alpha polypeptide 1 (muscle)	−1,5	−2,8
11811	*Apobec2*	apolipoprotein B mRNA editing enzyme, catalytic polypeptide 2	−1,5	−2,9
24131	*Ldb3*	LIM domain binding 3	−1,6	−3,0
17927	*Myod1*	myogenic differentiation 1	−1,6	−3,1
66139	*Tmem8c*	transmembrane protein 8C	−1,7	−3,2
21828	*Thbs4*	thrombospondin 4	−1,8	−3,4
140781	*Myh7*	myosin, heavy polypeptide 7, cardiac muscle, beta	−1,9	−3,7
58916	*Myot*	myotilin	−2,0	−3,9
87,201,087	*Tnnt1*	troponin T1, skeletal, slow	−2,0	−3,9
17928	*Myog*	myogenin	−2,1	−4,3
53311	*Mybph*	myosin binding protein H	−2,2	−4,5
21952	*Tnni1*	troponin I, skeletal, slow 1	−2,4	−5,3
11,472	*Actn2*	actinin alpha 2	−2,4	−5,4
17896	*Myl4*	myosin, light polypeptide 4	−2,5	−5,5
66,402	*Sln*	sarcolipin	−2,5	−5,5
21,380	*Tbx1*	T-box 1	−2,6	−6,1
21956	*Tnnt2*	troponin T2, cardiac	−2,6	−6,2
66106	*Smpx*	small muscle protein, X-linked	−2,7	−6,5
11464	*Actc1*	actin, alpha, cardiac muscle 1	−2,7	−6,5
92,760,598	*Tnnc1*	troponin C, cardiac/slow skeletal	−2,7	−6,6
21925	*Tnnc2*	troponin C2, fast	−2,8	−6,9
17901	*Myl1*	myosin, light polypeptide 1	−2,8	−7,2
17907	*Mylpf*	myosin light chain, phosphorylatable, fast skeletal muscle	−3,0	−7,9
80,608,559	*Tnnt2*	troponin T2, cardiac	−3,1	−8,5
11,459	*Acta1*	actin, alpha 1, skeletal muscle	−3,1	−8,7
17883	*Myh3*	myosin, heavy polypeptide 3, skeletal muscle, embryonic	−3,2	−9,3

Genes are listed based on fold change

**Table 2:** Table of genes originate from the comparison of Group A and Group C lists

Gene ID	Gene symbol	Description
Fifty-eight commonly expressed gene set list from Group A and Group C comparison		
16,545	*Acta1*	actin, alpha 1, skeletal muscle
11,475	*Acta2*	actin, alpha 2, smooth muscle, aorta
11,464	*Actc1*	actin, alpha, cardiac muscle 1
11,472	*Actn2*	actinin alpha 2
11,609	*Agtr2*	angiotensin II receptor, type 2
170,812	*Ahsp*	alpha hemoglobin stabilizing protein
11,811	*Apobec2*	apolipoprotein B mRNA editing enzyme, catalytic polypeptide 2
11,937	*Atp2a1*	ATPase, Ca++ transporting, cardiac muscle, fast twitch 1
59,058	*Bhlhe22*	basic helix-loop-helix family, member e22
12,299	*Cacng1*	calcium channel, voltage-dependent, gamma subunit 1
12,299	*Cav3*	caveolin 3
11,443	*Chrnb1*	cholinergic receptor, nicotinic, beta polypeptide 1 (muscle)
12,862	*Cox6a2*	cytochrome c oxidase subunit VIa polypeptide 2
12,955	*Cryab*	crystallin, alpha B
12,955	*Cryab*	crystallin, alpha B
13,009	*Csrp3*	cysteine and glycine-rich protein 3
50,768	*Dlc1*	deleted in liver cancer 1
50,796	*Dmrt1*	doublesex and mab-3 related transcription factor 1
14,066	*F3*	coagulation factor III
14,619	*Gjb2*	gap junction protein, beta 2
69,253	*Hspb2*	heat shock protein 2
56,069	*Il17b*	interleukin 17B
24,131	*Ldb3*	LIM domain binding 3
17,189	*Mb*	myoglobin
17,286	*Meox2*	mesenchyme homeobox 2
53,311	*Mybph*	myosin binding protein H
17,883	*Myh3*	myosin, heavy polypeptide 3, skeletal muscle, embryonic
140,781	*Myh7*	myosin, heavy polypeptide 7, cardiac muscle, beta
17,901	*Myl1*	myosin, light polypeptide 1
17,896	*Myl4*	myosin, light polypeptide 4
17,907	*Mylpf*	myosin light chain, phosphorylatable, fast skeletal muscle
17,928	*Myog*	myogenin
17,929	*Myom1*	myomesin 1
17,930	*Myom2*	myomesin 2
58,916	*Myot*	myotilin
56,012	*Pgam2*	phosphoglycerate mutase 2
19,400	*Rapsn*	receptor-associated protein of the synapse
56,437	*Rrad*	Ras-related associated with diabetes
50,795	*Sh3bgr*	SH3-binding domain glutamic acid-rich protein
66,402	*Sln*	sarcolipin
66,106	*Smpx*	small muscle protein, X-linked
12,180	*Smyd1*	myosin, heavy polypeptide 7, cardiac muscle, beta
6899	*Tbx1*	T-box 1
12,384	*Tbx15*	T-box 15
21,393	*Tcap*	titin-cap
21,828	*Thbs4*	thrombospondin 4
66,139	*Tmem8c*	transmembrane protein 8C
21,924	*Tnnc1*	troponin C, cardiac/slow skeletal
21,925	*Tnnc2*	troponin C2, fast
21,952	*Tnni1*	troponin I, skeletal, slow 1
21,953	*Tnni2*	troponin I, skeletal, fast 2
21,955	*Tnnt1*	troponin T1, skeletal, slow
21,956	*Tnnt2*	troponin T2, cardiac
21,956	*Tnnt2*	troponin T2, cardiac
21,957	*Tnnt3*	troponin T3, skeletal, fast
67,951	*Tubb6*	tubulin, beta 6 class V
66,894	*Wwp2*	WW domain containing E3 ubiquitin protein ligase 2
26,465	*Zfp146*	zinc finger protein 146
Thirty uniquely expressed gene set of Group A		
11,656	*Alas2*	aminolevulinic acid synthase 2, erythroid
72,713	*Angptl1*	angiopoietin-like 1
107,765	*Ankrd1*	ankyrin repeat domain 1 (cardiac muscle)
12,292	*Cacna1s*	calcium channel, voltage-dependent, L type, alpha 1S subunit
12,350	*Car3*	carbonic anhydrase 3
12,350	*Car3*	carbonic anhydrase 3
12,575	*Cdkn1a*	cyclin-dependent kinase inhibitor 1A (P21)
12,608	*Cebpb*	CCAAT/enhancer binding protein (C/EBP), beta
13,038	*Ctsk*	cathepsin K
227,929	*Cytip*	cytohesin 1 interacting protein
13,346	*Des*	desmin
13,380	*Dkk1*	dickkopf homolog 1 (Xenopus laevis)
13,426	*Dync1i1*	dynein cytoplasmic 1 intermediate chain 1
14,077	*Fabp3*	fatty acid binding protein 3, muscle and heart
27,999	*Fam3c*	family with sequence similarity 3, member C
14,462	*Gata3*	GATA binding protein 3
15,891	*Ibsp*	integrin binding sialoprotein
65,086	*Lpar3*	lysophosphatidic acid receptor 3
23,945	*Mgll*	monoglyceride lipase
23,945	*Mgll*	monoglyceride lipase
17,533	*Mrc1*	mannose receptor, C type 1
27,273	*Pdk4*	pyruvate dehydrogenase kinase, isoenzyme 4
244,954	*Prss35*	protease, serine 35
51,801	*Ramp1*	receptor (calcitonin) activity modifying protein 1
20,466	*Sin3a*	transcriptional regulator, SIN3A (yeast)
231,633	*Tmem119*	transmembrane protein 119
21,953	*Tnni2*	troponin I, skeletal, fast 2
22,004	*Tpm2*	tropomyosin 2, beta
76,757	*Trdn*	triadin
22,145	*Tuba4a*	tubulin, alpha 4A
Twenty uniquely expressed gene set of Group C		
11,303	*Abca1*	ATP-binding cassette, sub-family A (ABC1), member 1
11,733	*Ank1*	ankyrin 1, erythroid
11,870	*Art1*	ADP-ribosyltransferase 1
11,435	*Chrna1*	cholinergic receptor, nicotinic, alpha polypeptide 1 (muscle)
74,374	*Clec16a*	C-type lectin domain family 16, member A
76,501	*Commd9*	COMM domain containing 9
12,846	*Comt*	catechol-O-methyltransferase
80,904	*Dtx3*	deltex 3 homolog (Drosophila)
108,655	*Foxp1*	forkhead box P1
14,809	*Grik5*	glutamate receptor, ionotropic, kainate 5 (gamma 2)
56,538	*Klk11*	kallikrein related-peptidase 11
16,979	*Lrrn1*	leucine rich repeat protein 1, neuronal
17,286	*Meox2*	mesenchyme homeobox 2
17,927	*Myod1*	myogenic differentiation 1
67,405	*Nts*	neurotensin
18,155	*Pnoc*	prepronociceptin
212,127	*Proser1*	proline and serine rich 1
54,153	*Rasa4*	RAS p21 protein activator 4
21,384	*Tbx15*	T-box 15
21,812	*Tgfbr1*	transforming growth factor, beta receptor I

Genes are listed alphabetically

All genes described derived from the statistically significant groups (adj. *p* < 0.1)

### Confirmation of microarray data

**Fig. 5:**
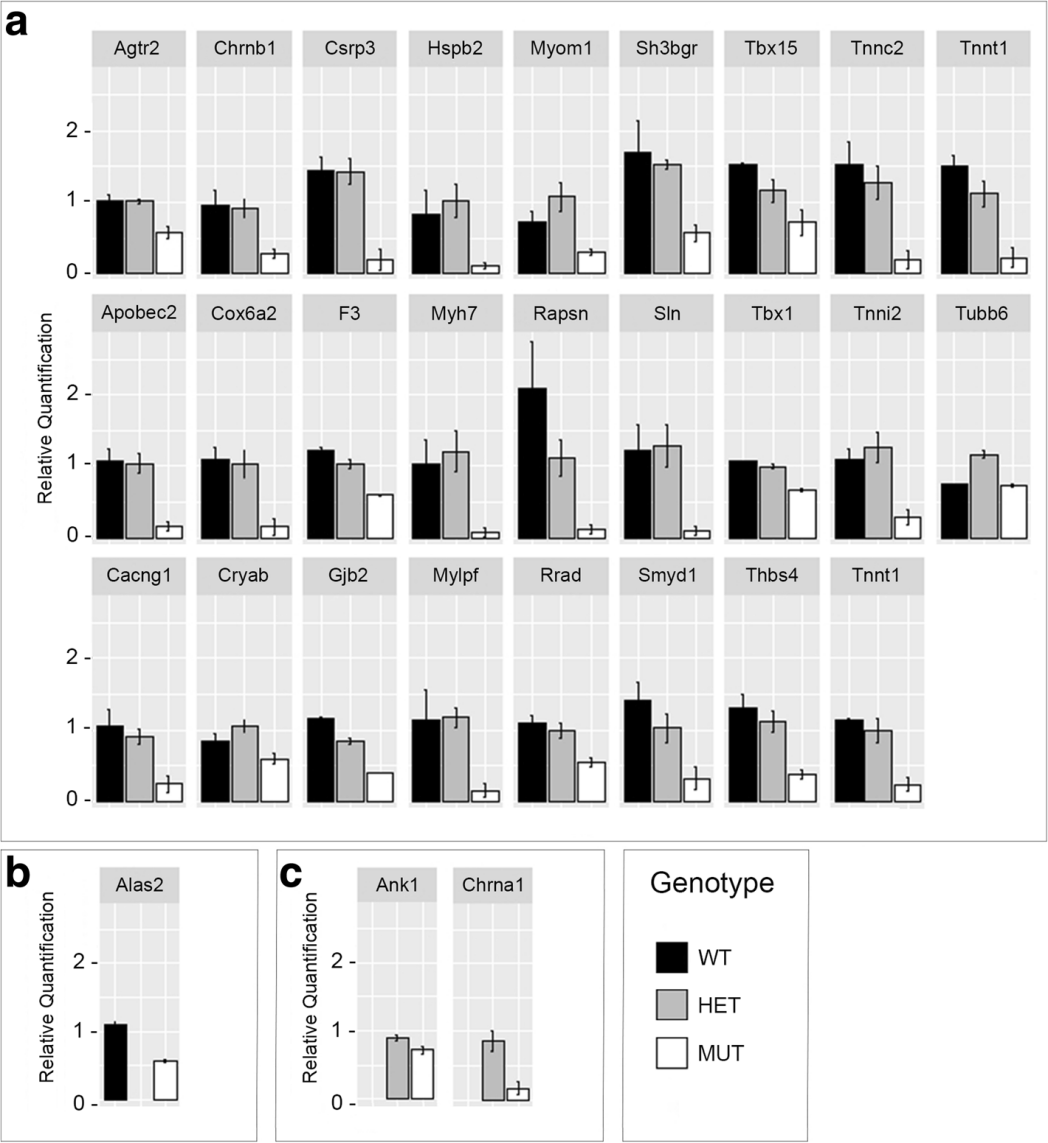
Quantitative reverse transcriptase polymerase chain reaction verification of genes identified in Groups A and C following the microarray analysis. **a** Common genes significantly changed between both WT-mutant and heterozygote-mutant; (**b**) genes significantly changed only between WT-mutant (Group A); (**c**) genes significantly changed only between heterozygote-mutant (Group C)

For validation of the results obtained by microarray, RT-PCR was carried out using gene-specific primers (Applied Biosystems; Additional file 4) and the original RNA samples. In total, 27 genes from Group A and 28 genes from Group C were selected for gene expression verification (Table 3). Changes in gene expression of these transcripts were normalized to that of ß-Actin. In both groups, 27 genes were commonly expressed (Table 3; Fig. 5a); *Alas2* was uniquely present in Group A, whereas *Ank1* and *Chrna1* were uniquely present in Group C (Table 3; Fig. 5b). All genes tested were confirmed as being significantly changed between WT-mutant and heterozygote-mutant except for *Ank1* (Group C; *p* = 0.102). In Group A, *Rapsn***,** *Sh3bgr*, *Tnnc2*, *Tnni2* and *Tnnt2* were the most downregulated genes; whereas in Group C, these were *Csrp3*, *Sh3bgr*, *Sln*, *Tnnc2*, *Tnni2*, *Myh7* and *Mylpf*.

**Table 3:** Validated genes from Groups A and C

Gene ID	Gene symbol	Description	Fold Change Group A	Fold change Group C	*P* Value Anova
Validated genes commonly expressed in Groups A and C					
69253	*Hspb2*	heat shock protein 2	−0.7	−0.94	0.0776
17907	*Mylpf*	myosin light chain, phosphorylatable, fast skeletal muscle	−1.1	− 1.15	0.053
140781	*Myh7*	myosin, heavy polypeptide 7, cardiac muscle, beta	−0.97	−1.24	0.0472
50795	*Sh3bgr*	SH3-binding domain glutamic acid-rich protein	−1.77	−1.41	0.0433
66402	*Sln*	sarcolipin	−1.25	− 1.36	0.0373
12955	*Cryab*	crystallin, alpha B	−0.28	− 0.56	0.0332
11443	*Chrnb1*	cholinergic receptor, nicotinic, beta polypeptide 1 (muscle)	−0.74	− 0.67	0.03
17929	*Myom1*	myomesin 1	−0.41	− 0.88	0.0299
12180	*Smyd1*	SET and MYND domain containing 1	−1.43	−0.79	0.0277
12299	*Cacng1*	calcium channel, voltage-dependent, gamma subunit 1	−0.91	− 0.69	0.0221
19400	*Rapsn*	receptor-associated protein of the synapse	−3.17	−1.08	0.0202
21925	*Tnnc2*	troponin C2, fast	−1.75	− 1.28	0.0187
21384	*Tbx15*	T-box 15	− 1.24	− 0.59	0.0176
56437	*Rrad*	Ras-related associated with diabetes	−0.69	− 0.51	0.0168
12862	*Cox6a2*	cytochrome c oxidase subunit VIa polypeptide 2	−1.01	−0.94	0.0132
21828	*Thbs4*	thrombospondin 4	−1.16	−0.87	0.0103
21953	*Tnni2*	troponin I, skeletal, fast 2	−1.75	− 1.28	0.00946
11811	*Apobec2*	apolipoprotein B mRNA editing enzyme, catalytic polypeptide 2	−0.97	− 0.94	0.00473
11609	*Agtr2*	angiotensin II receptor, type 2	−0.56	− 0.53	0.00368
21956	*Tnnt2*	troponin T2, cardiac	−1.67	− 1.01	0.00323
13009	*Csrp3*	cysteine and glycine-rich protein 3	−1.58	− 1.56	0.00302
67951	*Tubb6*	tubulin, beta 6 class V	−0.03	− 0.59	0.00251
21955	*Tnnt1*	troponin T1, skeletal, slow	−1.04	−0.82	0.00226
21380	*Tbx1*	T-box 1	− 0.80	−0.87	0.000242
14066	*F3*	coagulation factor III	−0.81	− 0.52	0.000234
14619	*Gjb2*	gap junction protein, beta 2	−0.92	− 0.48	0.00000341
Gene ID	Gene symbol	Description	Fold Change		*P* Value (t-test)
Validated gene uniquely expressed in Group A					
11656	*Alas2*	aminolevulinic acid synthase 2, erythroid	−0.65		0.0062
Validated genes uniquely expressed in Group C					
11,733	*Ank1*	ankyrin 1, erythroid	−0.21		0.102
11435	*Chrna1*	cholinergic receptor, nicotinic, alpha polypeptide 1 (muscle)	−0.69		0.025

Genes are listed based on *p* value

## Discussion

In the present study, functional explant assays and microarray analysis of gene expression was carried out in the palatal shelves of E13.5 mouse embryos WT, heterozygous or mutant for *Tbx1*. This was prompted by the knowledge that *Tbx1* is strongly expressed in epithelium of the palatal shelves throughout palatogenesis, mutant embryos demonstrate cleft palate with complete penetrance [23, 24, 47, 48] and the findings that *Tbx1* has multiple potential roles during normal palatal shelf elevation, elongation and adhesion [47, 48]. It is known that several regulatory networks underlie signaling between epithelium and mesenchyme during development of the secondary palate and we sought to discover potential genetic pathways disrupted during palatogenesis in the absence of *Tbx1*. We therefore focused our investigations at E13.5, just prior to the period of rapid growth and elevation [45].

A key finding of this profile is the association between an absence of *Tbx1* function and altered expression (primarily downregulation) in a number of muscle-related genes within the shelves of the secondary palate. Developing mononuclear and binucleate myofibril-containing skeletal muscle cells are identifiable within the palatal shelves at E13 [59] and findings of altered gene expression are perhaps not surprising, given the essential role of *Tbx1* during the development of branchiomeric musculature and somite-derived tongue muscles [60–62] and detectable expression in adult mouse muscle [63, 64]. In the embryo, Tbx1 activates the myogenic-determination genes myogenic factor 5 (*Myf5*) and myogenic differentiation (*MyoD*) in the mesodermal core of pharyngeal arches I and II [61]. In addition, loss of *Tbx1* results in impairment of the onset of myogenic specification [60] and Tbx1 synergizes with the myogenic factor Myf5 for initiation of myogenic cell fate [65]. Our array failed to identify variation in *Myf5* and *MyoD*, but verified downregulation of *Myf7* at E13.5 in mutant palatal shelves. This finding suggests that Tbx1 functions upstream of myosin heavy chain 7 (Myh7) during palatal shelf formation and just prior to elevation, possibly as a myogenic factor. The presence of asymmetric expression patterns of myogenic regulatory factors in early first arch-derived muscles of *Tbx1* mutant embryos might explain the absence of *Myf5* and *MyoD* gene transcripts [61]. In addition, both skeletal, smooth and non-muscle contractile systems have been identified and implicated in the process of normal palatal shelf elevation [66, 67]. A number of the downregulated genes identified have also been implicated in the process of skeletal and cardiac muscle contraction (*Tnni2, Tnnt1, Myh3, Myom1, Tnnc2*), which might reflect the lack of skeletal myogenic determination. Interestingly, microarray analysis of the early pharyngeal region of *Df1/*^*+*^*; Tbx1*^*+/−*^ embryos has previously demonstrated upregulation of *Tnnc2* [68]. It cannot be discounted that other intrinsic contractile systems might also be disrupted in the secondary palate of *Tbx1* mutant mice. Indeed, changes in expression levels were also identified in genes associated with intracellular calcium signaling (*Atp2a1*, *Tnnc2*, *Cacna1s*, *Tnnc1*), which is known to mediate a number of important physiological processes of relevance to palatogenesis, including skeletal and smooth muscle contraction, apoptosis, cell motility and proliferation [69].

After palatal shelf elevation, periderm cells joined by tight junctions are believed to function as a protective layer, preventing aberrant adhesions and playing an important role in mediating appropriate shelf adherence and epithelial differentiation [70, 71]. Loss of periderm is required at the tips of opposing palatal shelves and overall at sites where fusion is required [71]. In *Tbx1* mutant mice, aberrant oral adhesions between tongue and palatal shelves have been observed [48]. In the present study, the tight junction genes *Myh3*, *Mylpf*, *Myh7* and *Actn2* were downregulated in mutants at E13.5, suggesting a potential role for *Tbx1* in the normal function of tight junctions present within the palatal shelf epithelium.

Comparison between WT-mutant and heterozygous-mutant shelves revealed 58 genes commonly expressed in both groups. From these, 27 genes from Group A and 28 genes from Group C were selected for gene expression verification. Analysis revealed significant downregulation of 26 genes common to both groups (see Fig. 5a) with (*Alas2*) and (*Ank1, Chrna1*) individually downregulated in each group, respectively (see Fig. 5b). Statistical analysis revealed significant downregulation of all genes tested through RT-PCR with the exception of *Ank1* (*p* = 0.102; see Fig. 5b). Pathway analysis of these validated genes confirmed the associations between cardiac muscle contraction and calcium signaling, but also suggested links with dilated and hypertrophic cardiomyopathies. Although 22q11.2DS is commonly associated with conotruncal congenital heart defects, hypocalcemic dilated myocardiopathy has also been described in association with this condition [72]. RT-PCR validation of the microarray analysis demonstrated no significant changes in gene expression between WT and heterozygous shelves, consistent with the normal palatogenesis seen in heterozygous embryos [23].

Tbx1 is known to regulate both *Fgf8* and *Fgf10* expression in the early pharyngeal arches and cardiac outflow tract [64] and influence the spatial distribution of *Fgf8* and *Bmp4* in the early mandible [73]. It has also been suggested that *Fgf8* is significantly downregulated in the palatal shelf epithelium, whilst *Fgf10* is upregulated in the mesenchyme at E13.5 in *Tbx1* mutant embryos [47]. However, we found no evidence of altered transcript levels associated with these genes in our array. This same report also demonstrated diminished hyaluronic acid (HA) in the palatal shelves of *Tbx1* mutant mice and whilst we found no obvious genetic links to this finding within our array, HA has been shown to induce matrix metalloproteinase 9 (*MMP9*) [74], which was downregulated. However, whilst some members of the MMP family have been directly related to palatogenesis, at least in vitro; this did not include *MMP9* [75].

**Fig. 6:**
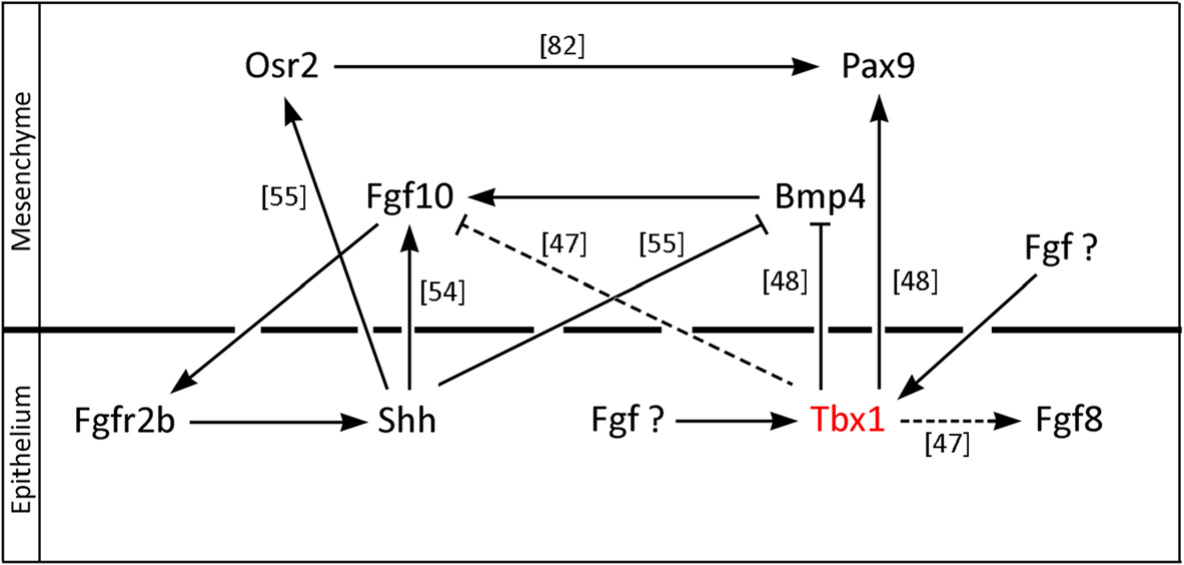
Molecular associations linking *Tbx1* with Fgf and Shh signaling in the developing palate. *Tbx1* in the palatal shelf epithelium is downstream of Fgf signaling, the ligand/s and source (epithelium/ mesenchyme) are currently unknown. Shh-Fgf10-Fgfr2b epithelial-mesenchymal reciprocal signaling [54] antagonizes *Bmp4* [55] and induces *Pax9* indirectly through the induction of *Osr2* [55, 82]. We and others [48] have demonstrated that Tbx1 acts to inhibit *Bmp4* and induce *Pax9*. It has been suggested that Tbx1 activity is required for *Fgf8* induction in the epithelium and *Fgf10* inhibition in the mesenchyme [47]; however, we and others [48] have found no evidence of this

In this microarray experiment, RNA was derived from whole dissected palatal shelves and therefore no formal distinction was made between changes in epithelial and mesenchymal gene activity. *Tbx1* is localized to the palatal shelf epithelium at E13.5, but is clearly able to influence signaling activity between epithelium and mesenchyme in the palate (Fig. 6). Indeed, the associations between Tbx1 function and muscle contraction and calcium signaling, both activities that take place in the early mesenchyme, are consistent with this. In addition, Tbx1 seems to act co-operatively with Shh signaling in the palate, through the repression of *Bmp4* and induction of *Pax9*. Interestingly, this co-operative activity would appear to be dependent upon Fgf signaling; *Shh* in the epithelium is dependent upon reciprocal signaling with Fgf10 in the mesenchyme [54] and our explant studies demonstrate that *Tbx1* is also dependent upon Fgf signaling. Although it is currently not known which Fgf ligand is required or whether this is within the epithelium or mesenchyme, maintenance of epithelial *Tbx1* transcription is essential for normal palatogenesis. Conditional loss of *Tbx1* in either craniofacial mesenchyme [48] or mesoderm [76] does not result in cleft palate, in contrast to loss-of-function in the oral epithelium, which does [48].

## Conclusions

We have conducted functional microarray analysis and PCR validation of gene expression in the developing secondary palate at E13.5 in the *Tbx1* mutant embryo. Differentially regulated genes were detected in the absence of this transcription factor. In the microarray, a total of 89 genes demonstrated differential expression in Group A and 88 genes in Group C (adj. *p* < 0.1), whilst high-throughput quantitative RT-PCR confirmed 27 genes significantly changed between WT and mutant and 28 between heterozygote and mutant. Associations existed with cardiac muscle development, hypertrophic and dilated cardiomyopathy, tight junction and calcium signaling. These findings provide further evidence of a primary role for *Tbx1* during the process of palatogenesis.

## Methods

### Mice

Breeding mice were maintained in ventilated cages on an alternating (12:12) light-dark cycle in the Biological Services Unit at King’s College London. Time-mated *Tbx1* embryos were generated by inter-crossing *Tbx1*^*+/−*^ mice on a C57/Bl6 background [23] such that noon of the day on which vaginal plugs were detected was considered as embryonic day (E) 0.5. Pregnant females were euthanized with cervical dislocation.

#### Explant culture

Secondary palatal shelves were carefully micro-dissected from E13.5 WT embryos and cultured for 24 h in the presence of cyclopamine or SU4502 as previously described [77]. Briefly, explants were cultured using a modified Trowell technique at 37 °C in an atmosphere of 5% CO2 in serum-free Advanced DMEM/F12 (GibcoBRL) supplemented with 20 U/ml penicillin and streptomycin (GibcoBRL), 10% Fetal Bovine Serum (GibcoBRL), 50 mM transferrin (Sigma) and 150 μg/ml ascorbic acid (Sigma). SU5402 (Calbiochem) was diluted in medium from a 10 mM stock solution in DMSO and cyclopamine (Sigma) was diluted from a 20 mg/ml stock solution in ethanol and added to the culture medium at a final concentration of 75 μM for both inhibitors. A minimum of (*n* = 6) palatal shelves were used for each experiment.

#### In situ hybridisation

Wholemount digoxygenin and section ^35^S radioactive in situ hybridisation was carried out as previously described [78]. Wholemount (*n* = 6 palatal shelves) and section (*n* = 3 embryos) images were photographed using Leica or Zeiss Axioscop microscopes, respectively. For radioactive in situ hybridisation, light and darkfield images were merged in Adobe photoshop CS. Plasmid cDNA was kindly provided by the following investigators: *Bmp4* (Brigid Hogan); *Fgf8* (Ivor Mason); *Fgf10; Fgfr2b* (David Rice); *Pax9* (Heiko Peters); *Ptch1* (Matthew Scott); *Shh* (Andy McMahon); *Sprty2* (M. Albert Basson), *Tbx1* (Peter Scambler).

#### Tissue preparation and microarray analysis

Secondary palatal shelves were carefully micro-dissected from E13.5 *Tbx1* WT, heterozygous or mutant embryos (3 embryos per genotype), stored as pairs from each embryo in RNA*later* (Ambion) and then homogenized using a blunt 20-guage needle to an RNase-free syringe. Total RNA was extracted from homogenate derived from each shelf pair using an RNeasy Isolation Kit (Qiagen). RNA quality was checked using an Agilent Bioanalyzer and quantified with spectrophotometry (NanoDrop ND-1000). In total, 9 sets of RNA were collected, each derived from paired secondary palatal shelves harvested from each embryonic genotype (giving 3 samples from each genotype).

#### Microarray chip processing and data analysis

The expression profiling analysis was carried out at the Franklin-Wilkins Building Genomics Facility, King’s College London. Total RNA was reverse-transcribed and cRNA generated using the MessageAmp II-Biotin Enhanced cRNA Amplification Kit (Ambion). cRNA targets were then hybridized to the Affymetrix Mouse GeneChip microarray (MOE430_A_2 GeneChip array), which is a single array containing 22,690 probe sets representing transcripts and variants from over 14,000 well characterized mouse genes. A single chip was used for each pair of palatal shelves per genotype, with hybridization and scanning of array chips carried out according to recommended protocols (www.affymetrix.com).

Microarray data were analysed by the implementation of Bioconductor packages in the programming language R. Intensity values of every chip were imported and evaluated with the packages *affy*, *simpleaffy* and *affyPLM*. Pre-processing, normalization and expression transformations were executed by the function *rma* of the *affy* package [79]. Gene expressions were fitted to linear models and moderated t-statistics were calculated for specific comparisons using *lmfit* and *eBayes* functions of the *limma* package [80]. *P*-values were adjusted for multiple testing with the Benjamini & Hochberg FDR method [81], implemented within the *topTable* function of the *limma* package. Venn diagram and heatmap showing hierarchical clustering with complete linkage scaled by genes were constructed using the packages *VennDiagram* and *gplots* respectively. Microarray datasets have been submitted to the Gene Expression Omnibus (GEO) at NCBI (GSE37904).

#### Functional annotation of differentially regulated gene sets

In this study WEB-based GEne SeT AnaLysis Toolkit (WebGestalt, http://www.webgestalt.org/option.php, version 05/20/2014) was utilized to perform functional enrichment analysis on the data sets containing genes from the *Tbx1*^−/−^ versus WT shelves comparison (Group A), the *Tbx1*^−/−^ versus *Tbx1*^+/−^ shelves comparison (Group C) and the commonly expressed gene set of Group A and Group C. For each gene set, WebGestalt used the hypergeometric test to evaluate functional enrichment against predefined categories collected from KEGG. Statistical analysis was performed according to the current default settings.

#### Validation with high throughput quantitative real-time RT-PCR and data analysis

Candidate genes were validated with high-throughput real time quantitative RT-PCR using the same nine total RNA samples from the microarray screen. RNA was converted to first-strand cDNA using the High Capacity RNA-to-cDNA kit (Applied Biosystems). Real time PCR assays were identified using Applied Biosystems UmapIt tool to map microarray probeset IDs to inventoried Taqman(r) assays. cDNA samples and assay master mixes were combined on 384-well real-time PCR plates (Applied Biosystems) using the Biomek FX liquid handling robot (Beckman Coulter). A total of nine 384-well plates were used. Each cDNA sample was combined with each gene primer sequence and replicated across four wells, giving four technical replicates for each PCR reaction. Each 384-well plate contained a column for water (no-template control) and ß-Actin (house-keeping gene/endogenous control for data normalization) with a 7900HT Quantitative PCR machine (Applied Biosystems) used for the PCR reaction. The qPCR data was analysed using RQ manager (Applied Biosystems) and Microsoft Excel. The RQ manager uses CT values from the qPCR reaction along with normalisation of the data to provide Relative Quantification (RQ) values (RQ = 2–ΔΔCT) for gene expression. For the 26 commonly expressed genes from Group A and C, ANOVA was used to detect statistically significant differences in Relative Quantification group means between WT, heterozygous and mutant genotypes. The differences in Relative Quantification for the uniquely expressed Alas2 in Group A and Ank1, Chrna1 genes between the WT and the MUT (Group C) were analysed by using t-test. All the above statistical analyses and graphs designs were performed in R. For the graphs, the ggplot2 package was used (see Fig. 5). From the 29 genes selected from microarray analysis, 28 individual genes showed significant changes in expression levels in the mutant compared to WT and/or heterozygote (*P* value < 0.05), whereas only *Agtr2* from Group C was shown to be non-statistically significant (*P* value = 0.102), in qPCR.

## Additional files

Additional file 1: *Tbx1 lacZ* reporter expression in the developing murine palate. (A) E12.5; (B) E13.5; (C) E14.5; (D) E15.5. *Tbx1* is expressed in epithelium of the primary (yellow arrowhead) and secondary palate (white arrowhead) with expression persisting in these regions during the process of fusion (orange and pink arrowheads, respectively). Expression is also seen in the maxillary incisor tooth germs (green arrowhead), maxillary molar tooth germs (red arrowhead) and palatal rugae (black arrows). (TIF 2146 kb)

Additional file 2: List of genes differentially expressed in WT compared to heterozygous palates (*n* = 400) (Group B). (XLSX 33 kb)

Additional file 3: KEGG pathway analysis. (XLSX 11 kb)

Additional file 4: Quantitative RT-PCR primer/probe list. This table contains a complete list of the 63 primers/ probes used in the real-time quantitative RT-PCR analysis of gene expression in the developing palate of *Tbx1* mice. (DOCX 90 kb)

## Abbreviations

22q11.2DS: 22q11.2 deletion syndrome
Bmp: Bone morphogenetic protein
CAFS: Conotruncal anomaly face
DGS: DiGeorge syndrome
Fgf: Fibroblast growth factor
Fgfr2b: Fibroblast growth factor receptor 2b
KEGG: Kyoto Encyclopedia of Genes and Genomes
*Myf5*: Myogenic factor 5
Myh7: Myosin heavy chain 7
*MyoD*: Myogenic differentiation
OFT: Outflow tract
Pax9: Paired box 9
Ptch1: Patched 1
RT-PCR: Reverse Transcription Polymerase Chain Reaction
Shh: Sonic hedgehog
Smad: Mothers against decapentaplegic homologue
Spry2: Sprouty 2
*TBX1*: Transcription factor-encoding T-Box 1
VCFS: Velocardiofacial syndrome
WT: Wild Type
