# Supplementary figures and images for: Gene expression profiling in the developing secondary palate in the absence of Tbx1 function
Source: BMC Genomics. 2018 Jun 4;19:429. doi: 10.1186/s12864-018-4782-y (PMC5987606; doi:10.1186/s12864-018-4782-y)

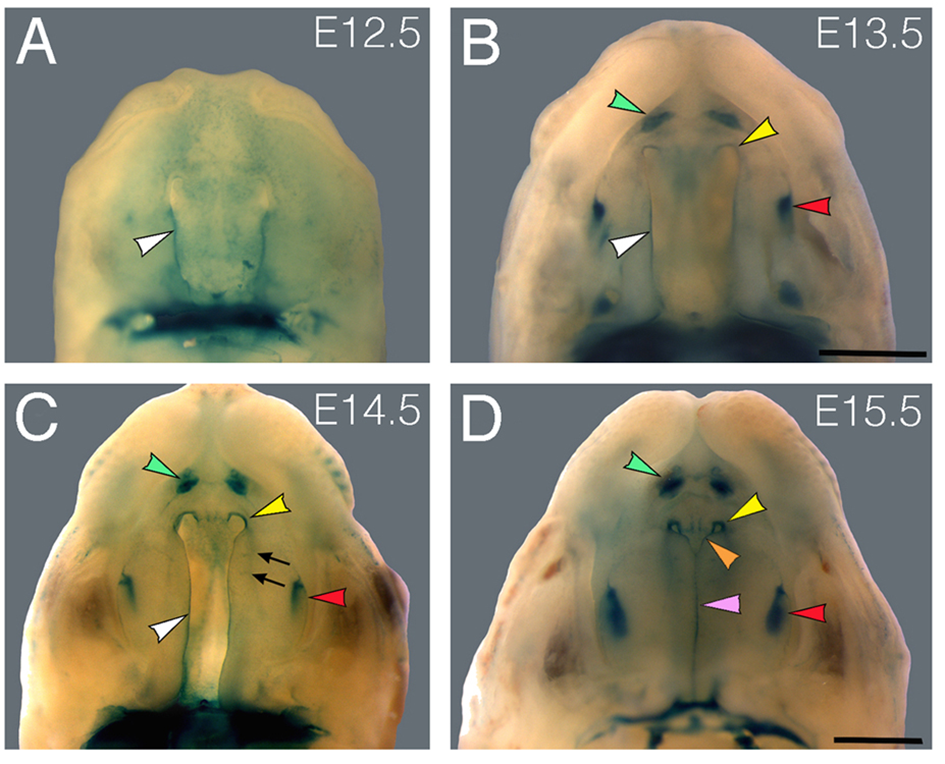

Supplement: Supplementary file 1 — Tbx1 lacZ reporter expression in the developing murine palate. (A) E12.5; (B) E13.5; (C) E14.5; (D) E15.5. Tbx1 is expressed in epithelium of the primary (yellow arrowhead) and secondary palate (white arrowhead) with expression persisting in these regions during the process of fusion (orange and pink arrowheads, respectively). Expression is also seen in the maxillary incisor tooth germs (green arrowhead), maxillary molar tooth germs (red arrowhead) and palatal rugae (black arrows). (TIF 2146 kb) [file 12864_2018_4782_MOESM1_ESM.tif]
