# Supplementary material for: Gene expression profiling in the developing secondary palate in the absence of Tbx1 function
Source: BMC Genomics. 2018 Jun 4;19:429. doi: 10.1186/s12864-018-4782-y (PMC5987606; doi:10.1186/s12864-018-4782-y)
Supplement: Supplementary file 4 — Quantitative RT-PCR primer/probe list. This table contains a complete list of the 63 primers/ probes used in the real-time quantitative RT-PCR analysis of gene expression in the developing palate of Tbx1 mice. (DOCX 90 kb) [file 12864_2018_4782_MOESM4_ESM.docx]

**Additional File 4**

**Zoupa et al.**

Quantitative RT-PCR primer/probe list. This table contains a complete list of the 63 primers/probes used in the real-time quantitative RT-PCR analysis of gene expression in the developing palate of *Tbx1* mice.

| **Gene symbol** | **Affymetrix® Probe set ID** | **TaqMan® Assay ID** |
| --- | --- | --- |
| Adamts8 | 1418270_at | Mm00479220_m1 |
| Adamtsl5 | 1420336_at | Mm00481701_m1 |
| Agtr2 | 1415832_at | Mm01341373_m1 |
| Akr1c13 | 1418672_at | Mm00657347_m1 |
| Alas2 | 1451675_a_at | Mm00802083_m1 |
| Ank1 | 1419421_at | Mm00482889_m1 |
| Apobec2 | 1417889_at | Mm00477588_m1 |
| Arpp21 | 1424248_at | Mm00473630_m1 |
| Cacnb4 | 1452089_at | Mm00521623_m1 |
| Cacng1 | 1422813_at | Mm00432246_m1 |
| Camk2b | 1455869_at | Mm00432296_m1 |
| Casq2 | 1422529_s_at | Mm00486742_m1 |
| Cbfb | 1460716_a_at | Mm01251026_g1 |
| Cbln1 | 1423287_at | Mm01247195_m1 |
| Ccl6 | 1420249_s_at | Mm00436446_g1 |
| Cd164 | 1431527_at | Mm00489798_m1 |
| Chrna1 | 1418852_at | Mm00431627_m1 |
| Chrnb1 | 1420682_at | Mm00680412_m1 |
| Cox6a2 | 1417607_at | Mm00438295_g1 |
| Cryab | 1434369_a_at | Mm00515567_m1 |
| Csrp3 | 1460318_at | Mm00443379_m1 |
| Epsti1 | 1452087_at | Mm00712734_m1 |
| F3 | 1417408_at | Mm00438855_m1 |
| Fam83f | 1424930_s_at | Mm00524379_m1 |
| Gjb2 | 1423271_at | Mm00433643_s1 |
| Glyat | 1427789_s_at | Mm00521549_m1 |
| Gnas | 1427789_s_at | Mm01242435_m1 |
| Gtf2i | 1431676_x_at | Mm00494826_m1 |
| Hist1h4h | 1428014_at | Mm00462640_s1 |
| Hspb2 | 1429888_a_at | Mm00517908_m1 |
| Ifit1 | 1450783_at | Mm00515153_m1 |
| Kif23 | 1450827_at | Mm00458527_m1 |
| Mmp9 | 1448291_at | Mm00442991_m1 |
| Myf5 | 1420757_at | Mm00435125_m1 |
| Myh3 | 1427115_at | Mm01332463_m1 |
| Myh7 | 1448553_at | Mm00600555_m1 |
| Mylpf | 1448371_at | Mm00443940_m1 |
| Myom1 | 1420693_at | Mm00440394_m1 |
| Ncam2 | 1425301_at | Mm00448056_m1 |
| Nacc1 | 1420968_at | Mm01251609_g1 |
| Olfr78 | 1421507_at | Mm00453733_s1 |
| Palmd | 1417251_at | Mm00652377_m1 |
| Rapsn | 1449331_a_at | Mm00485539_m1 |
| Rrad | 1422562_at | Mm00451053_m1 |
| Setd1a | 1427116_at | Mm00626141_m1 |
| Sgcg | 1421254_a_at | Mm00488741_m1 |
| Sh3bgr | 1422644_at | Mm00489429_m1 |
| Sln | 1420884_at | Mm00481536_m1 |
| Smyd1 | 1450203_at | Mm00477663_m1 |
| Sncb | 1418053_at | Mm00504325_m1 |
| Strn | 1422036_at | Mm00448910_m1 |
| Tbx15 | 1425779_a_at | Mm00447443_m1 |
| Tbx1 | 1422195_s_at | Mm00448948_m1 |
| Thbs4 | 1449388_at | Mm03003598_s1 |
| Tmem40 | 1424966_at | Mm00460633_m1 |
| Tnnc2 | 1417464_at | Mm00437116_m1 |
| Tnni2 | 1416889_at | Mm00437157_g1 |
| Tnnt1 | 1419606_a_at | Mm00449089_m1 |
| Tnnt2 | 1424967_x_at | Mm01290252_g1 |
| Tubb6 | 1416431_at | Mm00660543_m1 |
| Upb1 | 1460244_at | Mm00506060_m1 |
| Xiap | 1450231_a_at | Mm00776505_m1 |
| Xpr1 | 1437958_at | Mm00495501_m1 |
